# Demographics and Genetic Variability of the New World Bollworm (*Helicoverpa zea*) and the Old World Bollworm (*Helicoverpa armigera*) in Brazil

**DOI:** 10.1371/journal.pone.0113286

**Published:** 2014-11-19

**Authors:** Natália A. Leite, Alessandro Alves-Pereira, Alberto S. Corrêa, Maria I. Zucchi, Celso Omoto

**Affiliations:** 1 Departamento de Entomologia e Acarologia, Escola Superior de Agricultura “Luiz de Queiroz”, Universidade de São Paulo, Piracicaba, São Paulo, Brazil; 2 Departamento de Genética, Escola Superior de Agricultura “Luiz de Queiroz”, Universidade de São Paulo, Piracicaba, São Paulo, Brazil; 3 Agência Paulista de Tecnologia dos Agronegócios, Piracicaba, São Paulo, Brazil; Instituto de Higiene e Medicina Tropical, Portugal

## Abstract

*Helicoverpa armigera* is one of the primary agricultural pests in the Old World, whereas *H. zea* is predominant in the New World. However, *H. armigera* was first documented in Brazil in 2013. Therefore, the geographical distribution, range of hosts, invasion source, and dispersal routes for *H. armigera* are poorly understood or unknown in Brazil. In this study, we used a phylogeographic analysis of natural *H. armigera* and *H. zea* populations to (1) assess the occurrence of both species on different hosts; (2) infer the demographic parameters and genetic structure; (3) determine the potential invasion and dispersal routes for *H. armigera* within the Brazilian territory; and (4) infer the geographical origin of *H. armigera*. We analyzed partial sequence data from the cytochrome c oxidase subunit I (COI) gene. We determined that *H. armigera* individuals were most prevalent on dicotyledonous hosts and that *H. zea* were most prevalent on maize crops, based on the samples collected between May 2012 and April 2013. The populations of both species showed signs of demographic expansion, and no genetic structure. The high genetic diversity and wide distribution of *H. armigera* in mid-2012 are consistent with an invasion period prior to the first reports of this species in the literature and/or multiple invasion events within the Brazilian territory. It was not possible to infer the invasion and dispersal routes of *H. armigera* with this dataset. However, joint analyses using sequences from the Old World indicated the presence of Chinese, Indian, and European lineages within the Brazilian populations of *H. armigera*. These results suggest that sustainable management plans for the control of *H. armigera* will be challenging considering the high genetic diversity, polyphagous feeding habits, and great potential mobility of this pest on numerous hosts, which favor the adaptation of this insect to diverse environments and control strategies.

## Introduction

The Heliothinae (Lepidoptera: Noctuidae) subfamily has 381 described species, many of which are important agricultural pests from the *Helicoverpa* Hardwick and *Heliothis* Ochsenheimer genera [1]. The *Helicoverpa* genus contains two of the primary Heliothinae pest species: *Helicoverpa armigera* (Hübner) (Old World bollworm) and *Helicoverpa zea* (Boddie) (New World bollworm). Although the exact evolutionary relationship between *H. armigera* and *H. zea* remains uncertain, these insects are considered to be ‘twin’ or ‘sibling’ species, and they are able to copulate and produce fertile offspring under laboratory conditions [2]–[5]. Some hypotheses propose that *H. zea* evolved from a small portion of the larger *H. armigera* population (i.e., a “founder effect”) that reached the American continent approximately 1.5 million years ago, which is consistent with previous phylogeographic analyses of *H. armigera* and *H. zea* individuals [6], [7].

*H. armigera* is considered to be one of the most important agricultural pests in the world. This insect is widely distributed throughout Asia, Africa, Europe, and Australia, and it has been shown to attack more than 100 host species from 45 different plant families [8]–[10]. In contrast, *H. zea* is restricted to the American continent and is of lesser economic importance; it is a secondary pest of cotton, tomato, and, most significantly, maize crops [11]. However, the scenario in Brazil changed in 2013 when *H. armigera* individuals, which are considered to be A1 quarantine pests, were officially reported within the Brazilian territory [12]–[14]. This situation increased in severity due to the great dispersal ability of this insect as well as the steady reports from several regions of the world that described new *H. armigera* lineages showing tolerance/resistance to insecticides and genetically modified plants [15], [16]. It is estimated that *H. armigera* will cause a loss of more than US$2 billion to the 2013/14 Brazilian agriculture crop because of direct productivity losses and resources spent on phytosanitary products for soybean, cotton, and maize, which are the main crops of Brazilian agribusinesses. Therefore, *H. armigera* is now one of the most important pest species with respect to agriculture in Brazil [17].

High population densities of *Helicoverpa* spp. and the resulting economic damages to cultivated plants have been reported in different regions of Brazil, in particular in the Western state of Bahia [18]. Therefore, these reports suggest the existence of an invasion period prior to the first official report of *H. armigera* in Brazil. This atypical and confusing scenario was likely caused by the significant morphological similarities between *H. zea* and *H. armigera* [9], [19] and by major changes in pest management programs over recent years. In addition, these population changes may have been related to the release and increased cultivation of crops that express *Bacillus thuringiensis* (Bt) genes in Brazil.

Aside from the identification of *H. armigera* individuals within the Brazilian territory, many basic pieces of information concerning this species, including its geographical distribution, the types of hosts it attacks, its invasion source, and its dispersal routes, remain poorly understood or completely unknown. Therefore, we attempted to address some of these outstanding questions using a phylogeographic approach by analyzing genetic sequence data from a portion of the cytochrome c oxidase subunit I (COI) gene of *Helicoverpa* spp. specimens isolated from different hosts and regions of Brazil. This study was performed with the following goals in mind: (1) to confirm and evaluate the occurrence of *H. armigera* and *H. zea* individuals from different hosts and regions of Brazil; (2) to assess the demographic parameters and genetic structure of *H. armigera* and *H. zea* populations within the Brazilian territory, with a focus on the region, season, and host; (3) to assess the potential invasion (single or multiple) and dispersal routes for *H. armigera* within the Brazilian territory; and (4) to determine the geographical origin of the *H. armigera* populations present in Brazil. This information will be essential for understanding the genetic diversity and population dynamics of these pests as well as for guiding both immediate control strategies (legal and/or phytosanitary) and subsequent long-term integrated management programs for the *Helicoverpa* spp. complex in Brazil.

## Results

### Identification of *Helicoverpa* spp., hosts, and geographic locations

**Figure 1:**
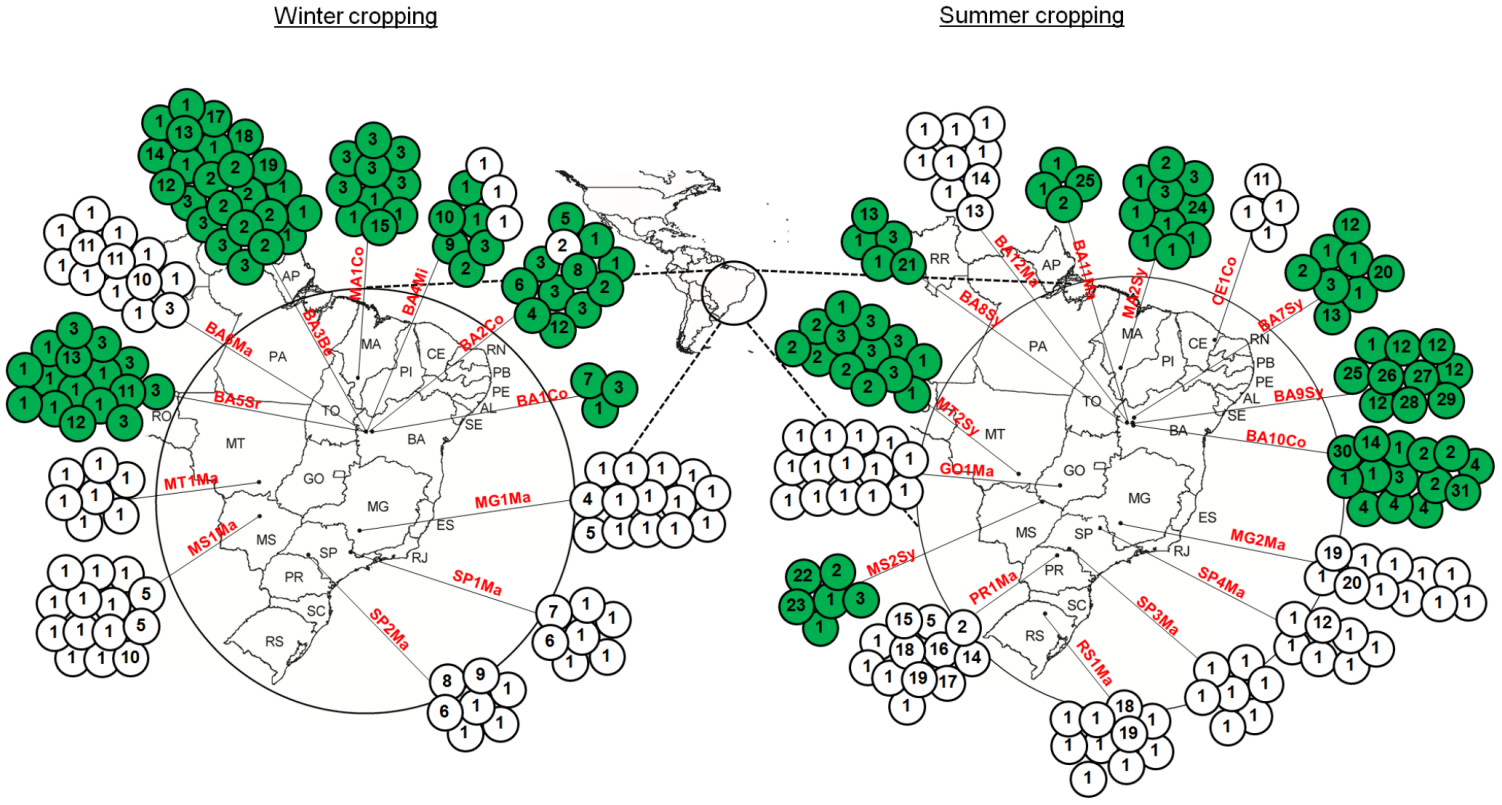
Geographic distributions of COI haplotypes of *H. armigera* and *H. zea*. One hundred and thirty nine and 135 COI haplotypes were analyzed for these species, respectively. The samples were separated into two temporal groups (winter crops and summer crops). Each circle represents the haplotypes identified in a given population; a number within a circle denotes the COI haplotypes identified in that population. Colored circles refer to *H. armigera* specimens, and white circles refer to *H. zea* specimens. The abbreviations refer to the sampled locations and crops (Table 1).

One hundred thirty-nine individuals from the 274 *Helicoverpa* spp. specimens initially sampled were identified as *H. armigera* (98–100% homology) and 134 individuals were identified as *H. zea* (98–100% homology) (GenBank Accession numbers KM274936–KM275209 are listed in Table 1). *H. armigera* was primarily found on soybean, bean, and cotton crops, and these insects were widely distributed throughout the Midwest and Northeast of Brazil during both crop periods (winter and summer) (Figure 1). *H. armigera* was also found on sorghum, millet, and maize crops. However, for maize, *H. armigera* individuals were only found at one site during the summer growing season in Northeastern Brazil (state of Bahia). *H. armigera* was not found on maize crops in the Midwest, Southeast, or South of Brazil. *H. zea* was primarily found on maize crops and was present in all sampled regions during both the winter and summer growing seasons. Of the winter crops, millet and cotton were exceptional in that they could simultaneously support *H. zea* and *H. armigera* (Figure 1). We found no correlations between specific *H. armigera* mitochondrial lineages (haplotypes) and specific hosts (Figure 1).

**Table 1:** Sampling sites for *Helicoverpa armigera* and *Helicoverpa zea* in Brazil, including the sites where these insects were sampled for this study, abbreviations, sample sizes for the mitochondrial genes (COI), crops sampled, geographic coordinates, dates sampled, and GenBank Accession.

Sites (City, State)	Abbreviation (Site, Crop)	Crop	Sample size		Lat. (S)	Lon. (W)	Date	GenBank Accession
			*H. armigera*	*H. zea*				
*Winter cropping*								
Barreiras, Bahia	BA1Co	Cotton	3	-	12°08′54′′	44°59′33″	05.22.12	KM274936–KM274938
Luís E. Magalhães, Bahia	BA2Co	Cotton	11	1	12°05′58″	45°47′54″	05.24.12	KM274939–KM274950
Balsas, Maranhão	MA1Co	Cotton	10	-	07°31′59″	46°02′06″	06.23.12	KM274987–KM274996
Luís E. Magalhães, Bahia	BA3Be	Bean	23	-	12°05′58″	45°47′54″	06.12.12	KM274979–KM274986, KM275038–KM275052
Luís E. Magalhães, Bahia	BA4Mi	Millet	6	3	12°05′58″	45°47′54″	05.10.12	KM274951–KM274959
Luís E. Magalhães, Bahia	BA5Sr	Sorghum	16	-	12°05′58″	45°47′54″	05.10.12	KM274960–KM274975
Capitólio, Minas Gerais	MG1Ma	Maize	-	14	20°36′17″	46°04′19″	06.08.12	KM274997–KM275010
Luís E. Magalhães, Bahia	BA6Ma	Maize	-	13	12°05′58″	45°47′54″	06.12.12	KM274976–KM274978, KM275053–KM275062
Itapira, São Paulo	SP1Ma	Maize	-	7	22°26′11″	46°49′20″	06.12.12	KM275011–KM275017
Assis, São Paulo	SP2Ma	Maize	-	7	22°39′40″	50°23′58″	06.15.12	KM275018–KM275024
São Gabriel do Oeste, Mato Grosso do Sul	MS1Ma	Maize	-	13	19°23′37″	54°33′49″	06.27.12	KM275025–KM275038
Rondonópolis, Mato Grosso	MT1Ma	Maize	-	7	16°28′17″	54°38′14″	08.01.12	KM275063–KM275069
*Summer cropping*								
Riachão das Neves, Bahia	BA7Sy	Soybean	8	-	12°08′54″	44°59′33″	10.21.12	KM275070–KM275077
Luís E. Magalhães, Bahia	BA8Sy	Soybean	5	-	12°05′58″	45°47′54″	10.31.12	KM275078–KM275082
Rondonópolis, Mato Grosso	MT2Sy	Soybean	13	-	16°28′17″	54°38′14″	11.08.12	KM275083–KM275092, KM275156–KM275158
Chapadão do Sul, Mato Grosso do Sul	MS2Sy	Soybean	6	-	18°46′44″	52°36′59″	11.29.12	KM275097–KM275102
Balsas, Maranhão	MA2Sy	Soybean	10	-	07°31′59″	46°02′06″	01.06.13	KM275103–KM275112
São Desidério, Bahia	BA9Sy	Soybean	10	-	12°21′08″	44°59′03″	01.15.13	KM275127–KM275136
Limoeiro do Norte, Ceará	CE1Co	Cotton	-	4	05°08′56′′	38°05′52′′	10.08.12	KM275093–KM275096
São Desidério, Bahia	BA10Co	Cotton	14	-	12°21′08″	44°59′03″	01.15.13	KM275147–KM275155, KM275202–KM275206
Cândido Mota, São Paulo	SP3Ma	Maize	-	7	22°44′46″	50°23′15″	01.14.13	KM275113–KM275119
Jardinópolis, São Paulo	SP4Ma	Maize	-	7	21°03′47″	47°45′05″	03.04.13	KM275120–KM275126
Barreiras, Bahia	BA11Ma	Maize	4	-	11°33′33″	46°19′47″	02.21.13	KM275137–KM275140
Luís E. Magalhães, Bahia	BA12Ma	Maize	-	9	12°05′58″	45°47′54″	03.28.13	KM275141–KM275146, KM275207–KM275209
Rolândia, Paraná	PR1Ma	Maize	-	12	23°19′13′′	51°29′01′′	01.24.13	KM275159–KM275170
Passo Fundo, Rio Grande do Sul	RS1Ma	Maize	-	10	28°16′08′′	52°37′15′′	01.30.13	KM275171–KM275180
Montividiu, Goiás	GO1Ma	Maize	-	10	17°19′19′′	51°14′51′′	02.05.13	KM275181–KM275190
Capitólio, Minas Gerais	MG2Ma	Maize	-	11	20°36′17″	46°04′19″	03.10.13	KM275191–KM275201
**Total**			**139**	**135**				

### Dataset assembly, haplotypes, and demographic analysis

Following alignment and editing, we were unable to identify indels or stop codons in the sequences from either species. However, using the most common haplotype for each species as a reference, eight non-synonymous substitutions were observed in 17 *H. armigera* individuals, and four non-synonymous substitutions were observed in eight *H. zea* individuals. However, considering the relatively high mutation rate reported for the COI gene in the *Helicoverpa* genus [20], as well the absence of indels and stop codons, it is unlikely that these sequences represent numts (nuclear mitochondrial DNA).

Twenty-six polymorphic sites were found among the 139 *H. armigera* individuals sampled, which yielded 31 haplotypes with a haplotype diversity (Hd) of 0.821 and a nucleotide diversity (Pi) of 0.0028. Sequence analysis of the 134 sampled *H. zea* individuals identified 19 polymorphic sites, which yielded 20 haplotypes with an Hd of 0.420 and a Pi of 0.0011 (Table 2). No significant differences in Hd or Pi were found for either species when the individuals were separated by growing season according to the sampled crops (Table 2). The results from Tajima's D test were only not significant for *H. armigera* individuals (*p* = 0.07) sampled on summer crops; however, Fu's Fs test was significant (*p*<0.01). The Tajima's D and Fu's Fs test results for both *H. armigera* and *H. zea* were negative and significant when the individuals were tested as a single group and when the individuals were split into groups based on the crop on which they were sampled (summer or winter; temporally). These results indicate an excess of low frequency polymorphisms and are consistent with either population expansion or purifying selection (Table 2). In addition, the model of sudden expansion [21] did not reject the hypothesis of expansion demographics for *H. armigera* (SSD = 0.0012, *p* = 0.48; Raggedness  = 0.0433, *p* = 0.61) or *H. zea* (SSD = 0.0002, *p* = 0.90; Raggedness  = 0.1492, *p* = 0.72).

**Table 2:** Number of individuals, haplotype designation, and genetic diversity for the sampled populations grouped according to geographical origin.

Group	N. Individuals (samples)	N. haplotypes	Distribution of Haplotypes (n)	Haplotype Diversity (Hd)	Nucleotide diversity (Pi)	Tajima's D test (*p* value)	Fu's Fs test (*p* value)
***H. armigera***							
**Pooled**	**139 (14)**	**31**	**-**	**0.821**	**0.0028**	**−1.729 (<0.01)**	**−26.361 (<0.01)**
Winter cropping	69 (6)	19	H1(22); H2(9); H3(21); H4(1); H5(1); H6(1); H7(1); H8(1); H9(1); H10(1); H11(1); H12(2); H13(2); H14(1); H15(1); H16(1); H17(1); H18(1); H19(1).	0.805	0.0028	−1.608 ( = 0.03)	−11.891 (<0.01)
Summer cropping	70 (8)	19	H1(22); H2(13); H3(11); H4(4); H12(4); H13(2); H14(1); H20(1); H21(1); H22(1); H23(1); H24(1); H25(2); H26(1); H27(1); H28(1); H29(1); H30(1); H31(1).	0.835	0.0028	−1.353 ( = 0.07)	−11.254 (<0.01)
***H. zea***							
**Pooled**	**135 (16)**	**20**	**-**	**0.420**	**0.0011**	**−2.190 (<0.01)**	**−22.912 (<0.01)**
Winter cropping	65 (8)	11	H1(50); H2(1); H3(1); H4(1); H5(3); H6(2); H7(1); H8(1); H9(1); H10(2); H11(2).	0.408	0.0009	−2.156 (<0.01)	−9.735 (<0.01)
Summer cropping	70 (8)	13	H1(53); H2(1); H5(1); H11(1); H12(1); H13(1); H14(2); H15(1); H16(1); H17(1); H18(2); H19(3); H20(2).	0.427	0.0012	−1.967 (<0.01)	−10.411 (<0.01)

### Statistical analysis of population structure

The results of the analysis of molecular variance (AMOVA) with two hierarchical levels showed that the greatest amount of total variation was accounted for by differences among individuals within populations: 92.89% for *H. armigera* (Φ_ST_ = 0.071) and 94.22% for *H. zea* (Φ_ST_ = 0.058) (Table S1). For the AMOVA with three hierarchical levels for *H. armigera*, the largest percentage of variation occurred within populations, separating individuals into groups by time (winter and summer crops; 93.17%, Φ_CT_ = 0.006; Φ_SC_ = 0.074; Φ_ST_ = 0.068), host group (mono- and dicotyledonous; 99.24%, Φ_CT_ = −0.01; Φ_SC_ = 0.018; Φ_ST_ = 0.007), and each host type (crop; 93.19%, Φ_CT_ = −0.042; Φ_SC_ = 0.105; Φ_ST_ = 0.068) (Table S1). The group separation for *H. armigera* was not significant for any of the three tested groups (*p*>0.10). The AMOVA with three hierarchical levels divided the *H. zea* individuals into groups by time (winter and summer crops), which showed a larger variation within populations (93.76%, Φ_CT_ = 0.010; Φ_SC_ = 0.052; Φ_ST_ = 0.062); the group division was not significant (*p>*0.10) (Table S1).

### Network analysis and Bayesian phylogeny

**Figure 2:**
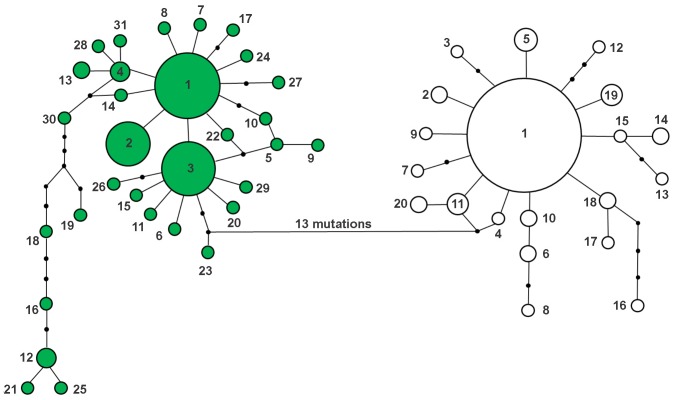
Haplotype network based COI sequences from *H. armigera* and *H. zea* samples collected in Brazil. Partial mtDNA COI (658 bp) sequences from *H. armigera* (colored circles) and *H. zea* (white circles) were analyzed from samples collected in Brazil. Each haplotype is represented by a circle and is identified by a number from 1–31. The *H. armigera* and *H. zea* COI haplotypes are shown as described in Table 2. The numbers of nucleotide substitutions between the haplotypes are indicated by black circles. The total number of nucleotide substitutions separating the *H. armigera* specimens from the *H. zea* specimens is shown.

Analysis of the genetic connections between the *Helicoverpa* spp. represented in the haplotype network revealed a close genetic relation between *H. armigera* and *H. zea*, which were separated by only 13 mutational steps (Figure 2). By separately analyzing the connections between the genetic haplotypes of each species, we inferred the existence of two predominant maternal lineages for *H. armigera*: H1 (31.65%) and H3 (23.02%), which were located at the center of the haplotype network. The other haplotypes of *H. armigera,* with the exception of haplotype H2 (15.83%), all had frequencies below 5%. Haplotypes H19, H18, H16, H12, H21, and H25 formed an outer cluster within the haplotype network of *H. armigera* (Figure 2). The haplotype network for *H. zea* revealed a genetic haplotype relationship with a single central high-frequency lineage (H1 = 76.30%) surrounded by low-frequency haplotypes (<5%) (Figure 2).

**Figure 3:**
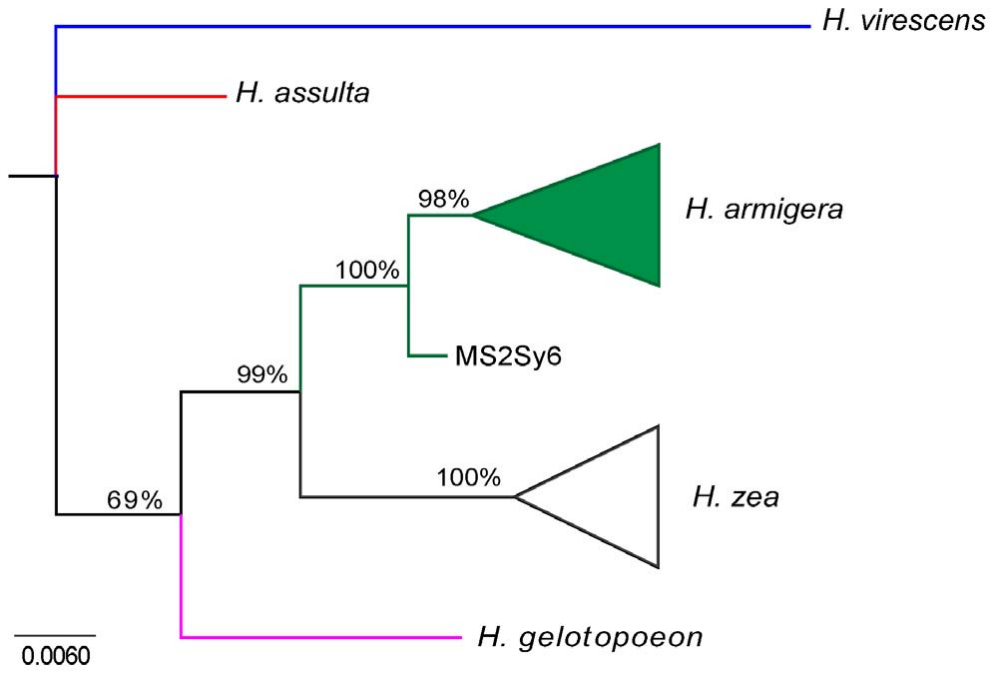
Bayesian phylogenetic tree of *H. armigera* and *H. zea* individuals sampled in Brazil. This phylogenetic tree is based on partial COI haplotype sequences and includes *H. assulta* and *H. gelotopoeon* sequences. Numbers near the interior branches indicate posterior probability (×100) values. The outgroup used was *Heliothis virescens*. *H. armigera* COI haplotypes and Genbank Accession numbers can be found in Table S2.

The optimal nucleotide substitution model identified by the MODELTEST 2.3 software program was the GTR+I+G model (Generalized time reversible + Proportion of invariable sites + Gamma distribution model). The estimated model parameters were based on empirical base frequencies (A = 0.3092, C = 0.1463, G = 0.1312, and T = 0.4133), with the proportion of invariable sites (I) set to 0.7393 and the gamma distribution shape parameter set to 0.5778. The consensus tree generated by the Bayesian analysis divided the *Helicoverpa* spp. specimens sampled in Brazil into two monophyletic clades (*H. armigera* and *H. zea*) with an associated probability of 99% (Figure 3; Figure S1). The probabilities separating the *H. zea* individuals into groups within this species were not significant. A single *H. armigera* individual (MS2Sy6) was separated from the other individuals with an associated probability of 98%. Finally, *Helicoverpa gelotopoeon* showed a closer phylogenetic relationship to *H. armigera* and *H. zea* compared with *H. assulta* (Figure 3; Figure S1).

### Network analysis: Brazilian vs. Old World *Helicoverpa armigera*

**Figure 4:**
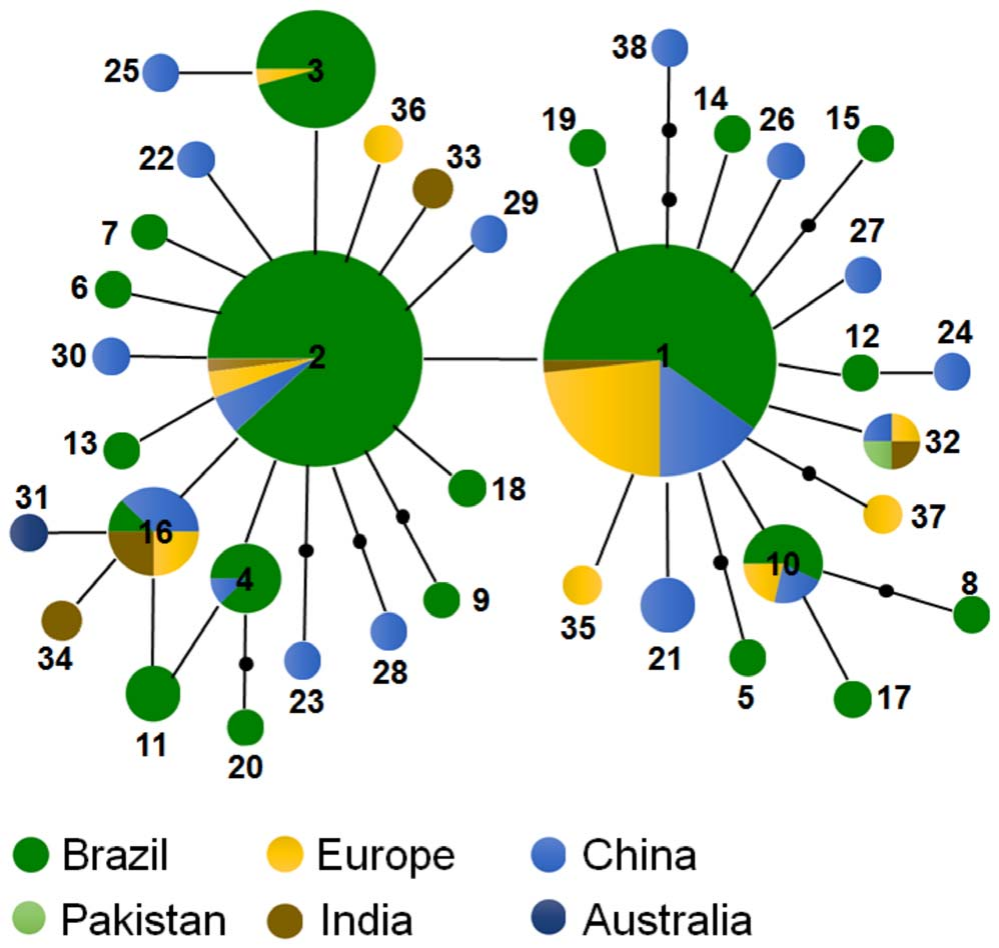
Haplotype network based COI sequences from *H. armigera* samples from Brazil and Old World specimens. Partial mtDNA COI (590 bp) sequences from this species were analyzed. Thirty-eight haplotypes were identified from 212 individuals sampled from China (n = 35), Thailand (n = 1), Australia (n = 1), Pakistan (n = 2), Europe (n = 28), India (n = 6), and Brazil (n = 139). *H. armigera* COI haplotypes are shown as described in Table S2. Each circle represents a haplotype and its number. The colors represent the frequency of each haplotype in the country/continent, with dark green (Brazil), light green (Pakistan), yellow (Europe), brown (India), light blue (China), and dark blue (Australia).

The haplotype network constructed using the edited sequences collected in Brazil, along with numerous Old World sequences, identified 38 distinct haplotypes (Figure 4). H1 (28%) and H2 (24%), which are widely distributed throughout Brazil, Europe, and China, were the most frequent haplotypes and occupied the central region of the haplotype network. All other haplotypes, with the exception of H3 and H10, showed frequencies below 5%. Finally, the majority of haplotypes with low frequencies represented by singletons were located at the network extremities (Figure 4).

## Discussion

Our results indicate a widespread distribution for *H. armigera* throughout the Midwest and Northeast of Brazil on a variety of crops, particularly dicotyledons, beans, soybeans, and cotton as well as, to a lesser extent, millet, sorghum, and maize. This pest was not found on maize crops in the Midwest, Southeast, or South of Brazil, despite the fact that these crops were initially identified as sources of *H*. *armigera* in this system. *H. armigera* individuals associated with maize crops were only found at a single sampling site in the Northeast (state of Bahia) during February 2013. In contrast, *H. zea* individuals were essentially found only on maize crops, with the exception of a few individuals collected from millet and cotton crops, where *H. zea* individuals were found alongside *H. armigera* individuals. Before the documentation of *H. armigera* in Brazil in 2013, we had hypothesized that major source of *Helicoverpa* spp. attacking different host plant was maize crops. However, our findings showed that targeting the control of *H. armigera* on maize crops may not be effective because *H. zea* was the predominant species in this host plant. The possibility of the formation of hybrid individuals between these two species, which has been reported under laboratory conditions [3], [4], needs to be investigated under field conditions to improve our pest management programs.

Demographic analyses using neutrality tests and a Mismatch Distribution Analysis indicated an expansion of the *H. armigera* and *H. zea* populations within the Brazilian territory. Population expansions were also consistent with the Haplotype network structure, which was characteristic of species undergoing processes of demographic expansion [22]. Brazilian *H. armigera* individuals showed two primary maternal lineages, whereas *H. zea* showed a single primary lineage, all of which were surrounded by numerous lower-frequency haplotypes. Therefore, these central high-frequency haplotypes represent the ancestral haplotypes, with the low-frequency haplotypes more recently derived [23]. Furthermore, signs of the *H. armigera* population expansion are likely because of the recent introduction of this pest into Brazil. Following the founder event, during which a portion of the overall genetic diversity of the species was introduced to Brazil, the *H. armigera* population further propagated. According to Nibouche et al. [24], *H. armigera* can migrate as far as 2,000 km, which likely facilitated the colonization of a variety of crops. The migration and colonization of crop areas by a small group of individuals can cause bottleneck effects, which, combined with plague population-suppression strategies (e.g., insecticide use that kills all but a small portion of the population), can lead to the types of demographic expansions observed for *H. zea* and *H. armigera* in Brazil [25]–[27]. In addition, the expansion of maize, soybean, and cotton crops into the North and Northeast of Brazil over the previous decade may also be responsible, in part, for the demographic expansion of these species, specifically *H. zea.* Additionally, assuming that not all COI variation is neutral, *Helicoverpa* spp. populations could be suffering selection, especially considering that populations have colonized new environments recently. However, further studies using a larger number of molecular markers from nuclear and mitochondrial genome regions would answer these questions. The *H. armigera* and *H. zea* population genetics were not structured according to space, time (winter and summer crops), or host (crops). Unstructured genetic networks have been reported for other populations of these two pest species in other parts of the world, which were based on several molecular markers, including mtDNA, allozymes, and microsatellites [7], [24], [25], [28], [29], [30]. Both species showed wide spatial haplotype distributions, and no genetic relationships were identified using a haplotype network analysis or an AMOVA. This scenario may be because these populations have a polyphagous feeding habit and migratory characteristics.

The unstructured population of *H. armigera* and the wide distribution of the two ancestral maternal lineages within the Brazilian territory did not allow us to infer any hypothetical invasion or dispersal routes for this species within the region. However, we noted that the haplotype and nucleotide diversities found for *H. armigera* in Brazil are similar to or greater than those reported for natural *H. armigera* populations in the Old World [7], [20]. For example, one outer branch of the *H. armigera* haplotype network, formed by haplotypes H19, H18, H16, H12, H21, and H25, is noteworthy for having the greatest genetic distance from the central haplotypes (H1 and H3), and these haplotypes have yet to be identified in Old World populations [7], [20]. In addition, joint analysis of the haplotypes from Brazil and the Old World yielded an overall structure that was similar to the haplotype network obtained only from the Brazilian individuals. In particular, the two most frequent haplotypes were identified throughout Brazil, Europe, China, and India, whereas the majority of the singletons were from Brazil and China. The cited literature, along with our results that showed a wide geographic distribution for *H. armigera* during the first half of 2012, support the hypothesis of an invasion period prior to the first reports of this species in Brazil. Alternatively, these findings are also consistent with a more recent invasion that involved a large gene pool, multiple invasion events, or some combination of these events.

The low genetic divergence observed between *H. armigera* and *H. zea* in the haplotype network analysis and the Bayesian phylogeny confirms the close genetic relatedness of these two species. Therefore, the reported co-occurrence of these species in time and space, as well as on the same hosts (as described here), could allow for the formation of hybrid individuals, which has been reported under laboratory conditions [3], [4]. Although the existence of hybrids in the wild remains unconfirmed, this scenario is of significant concern. In particular, recombination or introgression phenomena between *H. armigera*, which is reportedly resistant to control methods, and *H. zea*, which has adapted to the environmental conditions of the American continent, may enable gene transfer and fixation in some individuals. Therefore, hybridization may enable the selection of breeds with enhanced hybrid vigor and the ability to rapidly adapt to current management and suppression methods.

The population studies described in this study indicate a recent demographic expansion and a high mitochondrial genetic diversity for *H. armigera* and *H. zea* in Brazil. Therefore, the sustainable management of *H. armigera* will likely become a significant challenge for Brazilian entomology in the coming years, especially considering the polyphagous feeding habit, the great dispersal ability, and the numerous reports of resistance to insecticides and Bt crops for this insect [8], [24], [31]–[35]. This scenario requires immediate attention, as there is an imminent risk of *H. armigera* expanding throughout the American territory and perhaps reaching agricultural areas in Central and North America. However, it was not possible to trace the invasion and dispersal routes of *H. armigera* in the Brazilian territory. Nevertheless, the hypotheses of an invasion period prior to the first reports in the literature and/or an invasion that involved a diverse gene pool are both consistent with the observed high incidence and rapid adaptation of *H. armigera* in the Brazilian territory. Our confirmation that the predominant maternal lineages in the Brazilian territory are the same compared with those in Europe and Asia may represent a starting point to guide *H. armigera* management programs. Indeed, control strategies have a greater chance of success when reliable information is gathered in the regions where the pests, their hosts, and their natural enemies have co-evolved over a significant period of time.

## Materials and Methods

### Sampling procedures

Permit access to collect material used in our research at various crop sites was granted by respective growers. GPS coordinates of each location are listed in Table 1.

Brazilian agriculture has shown successive and overlapping crops in space and time, and these crops can be largely separated into two harvest groups that are primarily characterized by their rainfall needs. In particular, winter crops are grown between May and September and require low rainfall, whereas summer crops are grown between October and April and require high rainfall. Our initial sampling design was directed at understanding the *H. zea* population dynamics and primarily involved maize fields. However, attacks on soybean, cotton, bean, sorghum, and millet crops were also reported between May 2012 and April 2013 (Brazilian agricultural year). Therefore, we directed our sampling efforts towards a variety of crops and regions throughout Brazil. We also focused on the Western region of Bahia State, Brazil, which was the site of numerous *Helicoverpa* spp. attacks, to determine whether maize crops were the main source of *H. zea* in the Brazilian agricultural system. A total of 274 *Helicoverpa* caterpillars were collected at 19 sampling sites from six different crops (Table 1). In the absence of morphological characters or nuclear markers to reliably distinguish between *H. zea* and *H. armigera*, species identification was carried out using the sequence fragment of COI mitochondrial gene by comparing with *H. zea* and *H. armigera* species barcodes [7], [18], [19], [39] and determining homology with BlastN tool.

### DNA extraction, PCR amplification, and gene sequencing

Genomic DNA was isolated from the thorax of each adult using an Invisorb Spin Tissue Kit (STRATEC Molecular, Berlin, Germany), according to the manufacturer's protocol. A fragment of the COI mitochondrial gene was amplified by polymerase chain reaction (PCR) with the primers LCO(F) (5′ - GGT CAA CAA ATC ATA AAG ATA TTG G - 3′) and HCO(R) (5′ - TAA ACT TCA GGG TGA CCA AAA AAT CA - 3′) [36]. Amplification reactions were performed using 10 ng genomic DNA, 50 mM MgCl_2_, 0.003 mg.mL^−1^ BSA, 6.25 mM dNTPs, 10 pmol each primer, 1 U Taq DNA Polymerase (Life Technologies, Carlsbad, CA, USA), and 10% 10× Taq Buffer in a final volume of 25 µL. The PCR program consisted of an initial denaturation step at 94°C for 3 min, followed by 35 cycles of denaturation at 94°C for 30 s, annealing at 45°C for 30 s, and polymerization at 72°C for 1.5 min, with a final extension step at 72°C for 10 min. Following amplification, the aliquots were visually inspected using agarose gel (1.5% w/v) electrophoresis. The amplicons were purified by ethanol precipitation, and a second round of amplification was performed using the Big Dye Terminator v3.1 Cycle Sequencing system (Applied Biosystems, Foster City, CA, USA), which was followed by further purification. DNA sequencing was performed using the ABI3500xl automated genetic analyzer (Applied Biosystems, Foster City, CA, USA) at the State University of Campinas (Universidade Estadual de Campinas, Campinas, São Paulo, Brazil).

### Dataset assembly, haplotypes, and demographic analysis

All sequences were manually edited using the Chromas Lite version 2.01 [37] software program and were aligned using the ClustalW tool from the BioEdit version 7.0 [38] software program. After editing and aligning the COI sequences, we determined the 658 bp consensus sequence, which was then posteriorly compared with the *H. zea* and *H. armigera* species barcodes [41] to determine homology using the BlastN tool, which is available online at NCBI [40].

The MEGA version 4 [41] software program was used to inspect the COI sequences from each species individually for the presence of numts [42]. In particular, we searched for the following numt signatures: (i) insertions/deletions (*indels*); (ii) stop codons leading to premature protein termination; and (iii) increased rates of non-synonymous mutations. The presence of signatures (i) and (ii) was considered sufficient to regard a sequence as a COI numt. In the presence of signatures (i) or (ii), signature (iii) was used to confirm the sequence as a numt. The presence of signature (iii) alone was not considered sufficient to define a sequence as a numt.

Haplotype and nucleotide diversity parameters for each species were estimated using the DnaSP version 5 [43] software program. Neutrality tests using Tajima's D [44] and Fu's Fs [45] were performed using the Arlequin version 3.1 [46] software program, and significance was determined using 1,000 random samples in coalescent simulations. Based on the recommendations in the Arlequin manual, we activated the “Infer from distance matrix” option under “Haplotype definition”, and the Fu's Fs statistical values were considered to be significant at a level of 5% only when the *P*-value was below 0.02. The diversity estimates and neutrality tests were performed using all sampled individuals from each species, which were divided into winter-crop and summer-crop groups. A Mismatch Distribution Analysis using a spatial expansion model [21] was also performed using the Arlequin version 3.1 software program, and significance was determined using 1,000 bootstrap replicates. We used the goodness-of-fit of the observed mismatch distribution to the expected distribution from the spatial expansion model and the sum of square deviations (SSD) as a test statistic (*P*-value support).

### Population structure analysis

Using Arlequin 3.1, we also performed an AMOVA at the two- and three-hierarchy levels [47]. For the three-hierarchy AMOVA, we first separated the samples depending on whether they were collected on winter or summer crops and then further divided them by host plant (monocotyledonae or dicotyledonae).

### Network analysis and Bayesian phylogenies

Genetic differences and connections among *Helicoverpa* spp. haplotypes were determined by constructing a maximum parsimony network [48] using the TCS 1.21 software program [49]. To resolve ambiguities present in the haplotype network, we used the criteria of coalescence theory and population geography proposed by Crandall and Templeton [23].

We used the distance matrix option in the PAUP *4.0 software program to calculate the inter- and intra-species genetic distances, which were inferred using the nucleotide substitution model and the Akaike Information Criteria [50] selected by MODELTEST 2 [51]. The MrBayes v3.2 software program [52] was used to estimate Bayesian phylogenies. In particular, the Bayesian analysis was performed with 10 million generations using one cold and three heated chains. *Helicoverpa assulta* (Guenée) (GenBank Accession number: EU768937), *H. gelotopoeon* Dyar (EU768938), and *H. virescens* (IN799050) sequences were included as outgroups for the Bayesian analysis. We obtained a 50%-majority-rule consensus tree with posterior probabilities that were equal to the bipartition frequencies.

### Network analysis: Brazil vs. Old World

Seventy-three sequences from a variety of Old World sites that were present in GenBank were included with the 139 *H. armigera* sequences we collected in Brazil. In particular, 73 sequences were obtained from specimens collected in China (N = 35) [GenBank Accession numbers GQ892840 - GQ892855, GQ995232 - GQ995244 [20], HQ132369 (Yang, 2010), JX392415, and JX392497 (not published)], Thailand (1) [(EU768935)], Australia (1) [(EU768936) [5]], Pakistan (2) [(JN988529 and JN988530) (not published)], Europe (28) [(FN907979, FN907980, FN907988, FN907989, FN907996 - FN907999, FN908000 - FN908003, FN908005, FN908006, FN908011, FN908013 - FN908018, FN908023, FN908026, GU654969, GU686757, GU686955, and JF415782) (not published)] and India (6) [(HM854928-HM854932 and JX32104) (not published)] (Table S2). This new data set was edited and aligned as follows. The sequences were different lengths; thus, the editing and alignment processes generated a total of 212 sequences 590 bp in length, excluding indels. The sequences from individuals collected in Brazil, which were previously analyzed using a fragment length of 658 bp, as entered into GenBank (see Table 1), were edited by removing the first 36 bp and the last 32 bp. Using the TCS 1.21 software program [49], we subjected this data set to haplotype network analysis using a maximum parsimony network [48] to investigate the genetic connections between haplotypes from Brazil and the Old World as well as to infer the origins of maternal lineages within *H. armigera* populations in Brazil.

## Supporting Information

Figure S1 **Bayesian phylogenetic tree of** ***H. armigera*** **and** ***H. zea*** **individuals sampled in Brazil.** This phylogenetic tree is based on partial COI haplotype sequences and includes *H. assulta* and *H. gelotopoeon* sequences. Numbers near the interior branches indicate the posterior probability (×1,000) values. The outgroup used was *Heliothis virescens*. *H. armigera* COI haplotypes and Genbank Accession numbers can be found in Table S2.
(TIF)

Table S1 **Hierarchical analysis of molecular variance (AMOVA), for population genetics structure of** ***Helicoverpa armigera*** **and** ***H. zea*** **with a mithocondrial (COI) region marker.**
(DOCX)

Table S2 **Global** ***Helicoverpa armigera*** **including the Brazilian** ***H. armigera*** **haplotypes, and relevant GenBank Accession numbers. Numbers of individuals sequenced from each locality are indicated in parentheses.**
(DOCX)
