# Supplementary figures and images for: Demographics and Genetic Variability of the New World Bollworm (Helicoverpa zea) and the Old World Bollworm (Helicoverpa armigera) in Brazil
Source: PLoS One. 2014 Nov 19;9(11):e113286. doi: 10.1371/journal.pone.0113286 (PMC4237417; doi:10.1371/journal.pone.0113286)

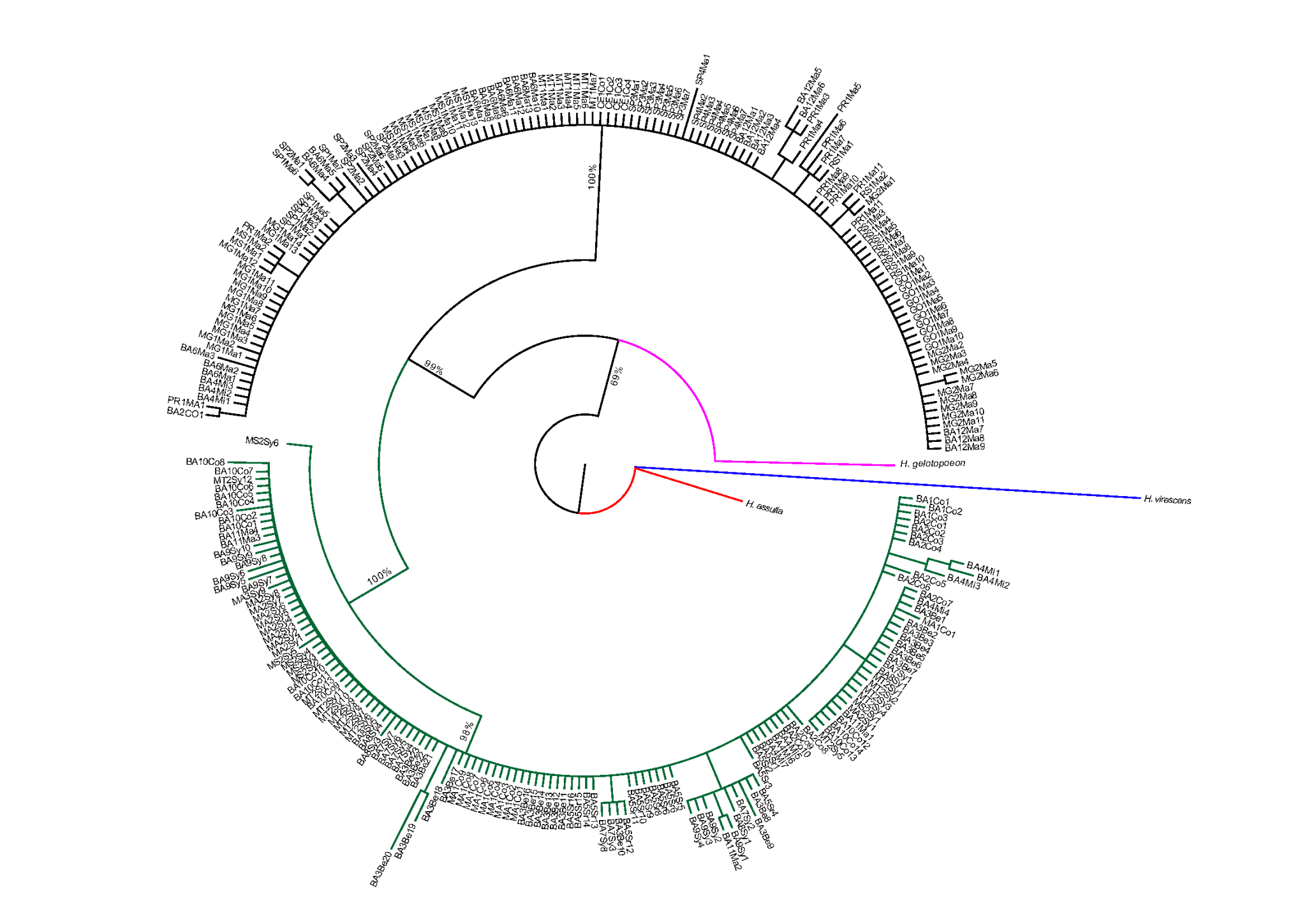

Supplement: Figure S1 — Bayesian phylogenetic tree of H. armigera and H. zea individuals sampled in Brazil. This phylogenetic tree is based on partial COI haplotype sequences and includes H. assulta and H. gelotopoeon sequences. Numbers near the interior branches indicate the posterior probability (×1,000) values. The outgroup used was Heliothis virescens. H. armigera COI haplotypes and Genbank Accession numbers can be found in Table S2. (TIF) [file pone.0113286.s001.tif]
