# Supplementary material for: Demographics and Genetic Variability of the New World Bollworm (Helicoverpa zea) and the Old World Bollworm (Helicoverpa armigera) in Brazil
Source: PLoS One. 2014 Nov 19;9(11):e113286. doi: 10.1371/journal.pone.0113286 (PMC4237417; doi:10.1371/journal.pone.0113286)
Supplement: Table S1 — Hierarchical analysis of molecular variance (AMOVA), for population genetics structure of Helicoverpa armigera and H. zea with a mithocondrial (COI) region marker. (DOCX) [file pone.0113286.s002.docx]

**Table S1.** Hierarchical analysis of molecular variance (AMOVA), for population genetics structure of *H. armigera* and *H. zea* with a mithocondrial (COI) region marker.

*Helicoverpa armigera*

| **Hierarchical levels** | **d.f.** | **Sum of Squares** | **Variance components** | **Variance (%)** | **Fixation Indices** | **P valor** |
| --- | --- | --- | --- | --- | --- | --- |
| **Two-hierarchical-levels** |  |  |  |  |  |  |
| Among populations | 15 | 21.897 | 0.0669Va | 7.11 | Φ_ST_=0.071 | <0.01 |
| Within populations | 127 | 111.019 | 0.8741Vb | 92.89 |  |  |
| Total | 142 | 132.916 | 0.9411 |  |  |  |
| **Three-hierarchical-levels (winter x summer cropping)** |  |  |  |  |  |  |
| Among groups | 1 | 1.276 | -0.0062Va | -0.66 | Φ_CT_=0.006 | =0.64 |
| Among populations within groups | 14 | 20.621 | 0.0703Vb | 7.49 | Φ_SC_=0.074 | <0.01 |
| Within populations | 127 | 111.019 | 0.8741Vc | 93.17 | Φ_ST_=0.068 | <0.01 |
| Total | 142 | 132.916 | 0.9382 |  |  |  |
| **Three-hierarchical-levels (di x mono)** |  |  |  |  |  |  |
| Among groups | 1 | 0.807 | -0.01056Va | -1.11 | Φ_CT_=-0.011 | =0.79 |
| Among populations within groups | 4 | 5.304 | 0.01783Vb | 1.87 | Φ_SC_=0.018 | =0.05 |
| Within populations | 133 | 125.522 | 0.94378Vc | 99.24 | Φ_ST_=0.007 | =0.12 |
| Total | 138 | 131.633 | 0.95104 |  |  |  |
| **Three-hierarchical-levels (six crops)** |  |  |  |  |  |  |
| Among groups | 5 | 6.111 | -0.04007Va | -4.21 | Φ_CT_=-0.042 | =0.74 |
| Among populations within groups | 9 | 15.503 | 0.10492Vb | 11.02 | Φ_SC_=0.105 | <0.01 |
| Within populations | 124 | 110.019 | 0.88725Vc | 93.19 | Φ_ST_=0.068 | <0.01 |
| Total | 138 | 131.633 | 0.95210 |  |  |  |

*Helicoverpa zea*

| **Hierarchical levels** | **d.f.** | **Sum of Squares** | **Variance components** | **Variance (%)** | **Fixation Indices** | **P valor** |
| --- | --- | --- | --- | --- | --- | --- |
| **Two-hierarchical-levels** |  |  |  |  |  |  |
| Among populations | 13 | 7.081 | 0.02111Va | 5.78 | Φ_ST_=0.0578 | <0.025 |
| Within populations | 121 | 41.637 | 0.34411Vb | 94.22 |  |  |
| Total | 134 | 48.719 | 0.36522 |  |  |  |
| **Three-hierarchical-levels (winter x summer cropping)** |  |  |  |  |  |  |
| Among groups | 1 | 0.826 | 0.00391Va | 1.07 | Φ_CT_=0.010 | =0.127 |
| Among populations within groups | 12 | 6.255 | 0.01897Vb | 5.17 | Φ_SC_=0.052 | <0.025 |
| Within populations | 121 | 41.637 | 0.34411Vc | 93.76 | Φ_ST_=0.062 | <0.023 |
| Total | 134 | 48.719 | 0.36700 |  |  |  |
