# Supplementary material for: Demographics and Genetic Variability of the New World Bollworm (Helicoverpa zea) and the Old World Bollworm (Helicoverpa armigera) in Brazil
Source: PLoS One. 2014 Nov 19;9(11):e113286. doi: 10.1371/journal.pone.0113286 (PMC4237417; doi:10.1371/journal.pone.0113286)
Supplement: Table S2 — Global Helicoverpa armigera including the Brazilian H. armigera haplotypes, and relevant GenBank Accession numbers. Numbers of individuals sequenced from each locality are indicated in parentheses. (DOCX) [file pone.0113286.s003.docx]

**Table S2:** Global *Helicoverpa armigera* including the Brazilian *H. armigera* haplotypes, and relevant GenBank Accession numbers. Numbers of individuals sequenced from each locality are indicated in parentheses.

| **Countries or Continents** | **Locations** | **COI Haplotypes** | **GenBank Accession numbers** |
| --- | --- | --- | --- |
| China | Kunming (8) | H1, H2, H3, H16, H21, H26, H30 | GQ892840.1, GQ892842.1, GQ892854.1, GQ995232.1, GQ995234.1, GQ995235.1, GQ995244.1, GQ995239.1 |
|  | Tibet (2) | H2, H10 | JX392497.1, JX392415.1 |
|  | Miaofengshan (4) | H1, H10, H32 | JX509766.1, JX509765.1, JX509764.1, JX509739.1 |
|  | Not specified (1) | H38 | HQ132369.1 |
|  | Dali (15) | H1, H2, H4, H16, H21, H22, H23, H24, H25, H27, H28, H29 | GQ892846.1, GQ892847.1, GQ892848.1, GQ892849.1, GQ892850.1, GQ892851.1, GQ892852.1, GQ892853.1, GQ995233.1, GQ995236.1, GQ995237.1, GQ995240.1, GQ995241.1, GQ995242.1, GQ995243.1 |
|  | Henan (2) | H1, H16 | GQ995238.1, GQ892855.1 |
|  | Lijiang (1) | H21 | GQ892845.1 |
|  | Yuxi (2) | H1, H21 | GQ892843.1, GQ892844.1 |
| Europe | Not specified (24) | H1, H2, H3, H10, H16, H32, H35, H36, H37 | FN907979.1, FN907980.1, FN907988.1, FN907989.1, FN907995.1, FN907996.1, FN907997.1, FN907998.1, FN907999.1, FN908000.1, FN908001.1, FN908002.1, FN908003.1, FN908005.1, FN908006.1, FN908011.1, FN908013.1, FN908014.1, FN908015.1, FN908016.1, FN908017.1, FN908018.1, FN908023.1, FN908026.1 |
|  | Germany (4) | H1, H3, H16 | GU654969.1, GU686757.1, GU686955.1, JF415782.1 |
| Australia | Toowoomba (1) | H1 | EU768936.1 |
|  |  |  |  |
|  |  |  |  |
|  |  |  |  |
| Tailand | Not specified (1) | H2 | EU768935.1 |
| India | Not specified (6) | H1, H16, H32, H33, H34 | HM854928.1, HM854929.1, HM854930.1, HM854931.1, HM854932.1, JX532104.1 |
| Pakistan | Not specified (2) | H29, H32 | JN988529.1, JN988530.1 |
| Brazil | Barreiras, Bahia (7) | H1, H2, H3, H6, H17 | KM274936-KM274938, KM27513 -KM275140 |
|  | Luís Eduardo Magalhães, Bahia (61) | H1, H2, H3, H4, H5, H7, H8, H9, H10, H11, H13, H20 | KM274939 -KM274941, KM274943- KM274950, KM274951-KM274953, KM274957-KM274975, KM274979 - KM274986, KM275038-KM275052, KM275078-KM275082 |
|  | Riachão das Neves, Bahia (8) | H1, H2, H3, H10, H11, H14 | KM275070-KM275077 |
|  | São Desidério, Bahia (24) | H1, H2, H3, H4, H10, H17, H18, H19 | KM275127-KM275136 |
|  | Balsas, Maranhão (20) | H1, H2, H3, H12, H16 | KM274987-KM274996, KM275103-KM275112 |
|  | Rondonópolis, Mato Grosso (13) | H1, H2, H3 | KM275083 -KM275092, KM275156-KM275158 |
|  | Chapadão do Sul, Mato Grosso do Sul (6) | H1, H2, H3, H15 | KM275097 -KM275102 |
